# A Narrative Synthesis of the Health Systems Factors Influencing Optimal Hypertension Control in Sub-Saharan Africa

**DOI:** 10.1371/journal.pone.0130193

**Published:** 2015-07-15

**Authors:** Juliet Iwelunmor, Jacob Plange-Rhule, Collins O. Airhihenbuwa, Chizoba Ezepue, Olugbenga Ogedegbe

**Affiliations:** 1 Department of Kinesiology and Community Health, University of Illinois, Urbana-Champaign, United States of America; 2 School of Medical Sciences, College of Health Sciences, Kwame Nkrumah University of Science and Technology, Kumasi, Ghana; 3 Department of Biobehavioral Health, The Pennsylvania State University, University Park, PA, United States of America; 4 Department of Neurology, Georgia Regents University, Augusta, GA, United States of America; 5 Center for Healthful Behavior Change, Division of General Internal Medicine, Department of Medicine, New York University Langone Medical Center, New York, New York, United States of America; Duke University Medical Center, UNITED STATES

## Abstract

**Introduction:**

In sub-Saharan Africa (SSA), an estimated 74.7 million individuals are hypertensive. Reducing the growing burden of hypertension in sub-Saharan Africa will require a variety of strategies one of which is identifying the extent to which actions originating at the health systems level improves optimal management and control.

**Methods and Results:**

We conducted a narrative synthesis of available papers examining health systems factors influencing optimal hypertension in SSA. Eligible studies included those that analyzed the impact of health systems on hypertension awareness, treatment, control and medication adherence. Twenty-five articles met the inclusion criteria and the narrative synthesis identified the following themes: 1) how physical resources influence mechanisms supportive of optimal hypertension control; 2) the role of human resources with enabling and/or inhibiting hypertension control goals; 3) the availability and/or use of intellectual resources; 4) how health systems financing facilitate and/or compromise access to products necessary for optimal hypertension control.

**Conclusion:**

The findings highlight the need for further research on the health systems factors that influence management and control of hypertension in the region.

## Introduction

In sub-Saharan Africa (SSA), there are an estimated 926 million people, of which an estimated 74.7 million individuals are hypertensive [1]. Detection and management of hypertension in the region remains suboptimal [2] so much so that by the year 2025, the number of hypertensive individuals is projected to increase by 68% to 125.5 million individuals [1]. While an integral part of optimal management includes addressing patient-level factors such as individual knowledge or awareness of hypertension [3], we focus exclusively on health system factors to determine the extent to which actions originating at this level-such access to drugs or health insurance coverage, influence hypertension control [4]. Moreover, while the patient-level factors have been explored by other studies [5–8], studies examining the health systems factors influencing hypertension awareness, treatment, control, and medication adherence in the region are scare. Also in high/middle income countries, although it has been suggested that health care systems present barriers to optimal hypertension control [9], available studies indicated that the narrow health services perspective often used in these countries may not be applicable to sub-Saharan African settings [10,11]. Thus, there is an urgent need to understand how factors operating at the health system level in SSA influence the quality and use of appropriate strategies focused on prevention and management hypertension.

Globally, the past decade has witnessed recognition of the need to strengthen health systems. From the publication of the World Health Report published in 2000 which focused on improving performance in health systems to the role of systems thinking for health systems strengthening, researchers and health organizations such as the World Health Organization increasingly suggested that effective health systems are a prerequisite for achieving good health outcomes. Recent examples of studies in which researchers explored the influence of health systems on health care in SSA include: health systems strengthening through insurance subsidies in Rwanda [12], disease control priorities and health systems performance in South Africa [13]; equity in financing and use of health care in Ghana, South Africa and Tanzania [14]; health systems effectiveness with the delivery of intermittent preventive treatment with sulphadoxine-pyrimethamine to pregnant women in Mali [15]; African leadership for sustainable policy and systems research [16]; improving health information systems across five sub-Saharan African countries (Ghana, Mozambique, Rwanda, Tanzania, and Zambia) [17], electricity access in healthcare facilities[18]; human resources for health [19] as well as the deployment of community health workers across rural sub-Saharan Africa[20].

One effort to define health systems and related characteristics was carried out by the WHO. In their seminal book entitled “Strengthening Health Systems to Improve Health Outcomes,” the World Health Organization [21] defined health systems as *‘all the organizations*, *people*, *and actions whose primary intent is to promote*, *restore*, *or maintain health through efforts that influence the determinants of health as well as direct health-improving activities*.*’* The WHO [21] also suggested that regardless of how they are organized, health systems must carry out basic functions known as the *‘six essential building blocks of a health system’* and they include: provide services; develop health workers; provide equitable access to essential medical products, vaccines, and technologies; ensure the use of reliable and timely information on health determinants, health systems performance and status; mobilize and allocate finances; and ensure health system leadership and governance.

However, there are sharp criticisms of the building blocks. Mounier-Jack and colleagues [22], highlighted the advantages and limitations of using the building blocks in applied research. The authors concluded that despite its value with creating common language and shared understanding, it falls short with evaluating the impact of specific interventions on health systems [22]. Similarly, Frenk [23] highlighted the need to move beyond common generalizations about health systems, including the perspective that all health systems can be *“defined as a mere list of different organizations or persons that participate in producing health services*.*”* Such a definition, he argues, ignores for example, the population which is an essential part of the system [23].

Despite these shortcomings, there is a lack of health systems analysis as applied to hypertension in SSA using either the WHO building blocks or any other model. Instead, recommended actions to improve health systems in SSA often include the need to strengthen health financing [24] and/or removal of user fees [25,26]. However, it is unknown whether other factors and not only user fees or adequate financing alone, play a role with facilitating or hampering access to health systems services that are necessary for achieving optimal hypertension control [25,26]. Given the impact of hypertension, projected to become the most consequential disease burden by 2025 in SSA, the aim of this paper is to critically examine the health systems factors influencing hypertension awareness, treatment, control, and medication adherence in the region.

## Methods

### Design

Using Popay and colleagues [27] as a guide, we conducted a narrative synthesis of available papers that examined the health systems factors influencing optimal management of hypertension in SSA. Narrative synthesis refers to ‘*an approach to the systematic review and synthesis of findings from multiple sources and relies primarily on the use of words and text to summarize and explain the findings of the synthesis’* [27]. It is used when statistical meta-analysis or another specialist form of synthesis (such as meta-ethnography for qualitative studies) is not feasible particularly due to extreme heterogeneity in the methodological descriptions of available studies [27]. A narrative synthesis was appropriate for this review given an initial scoping exercise that revealed that the literature was too heterogeneous to permit a meta-analysis [4].

### Data Sources

**Fig 1:**
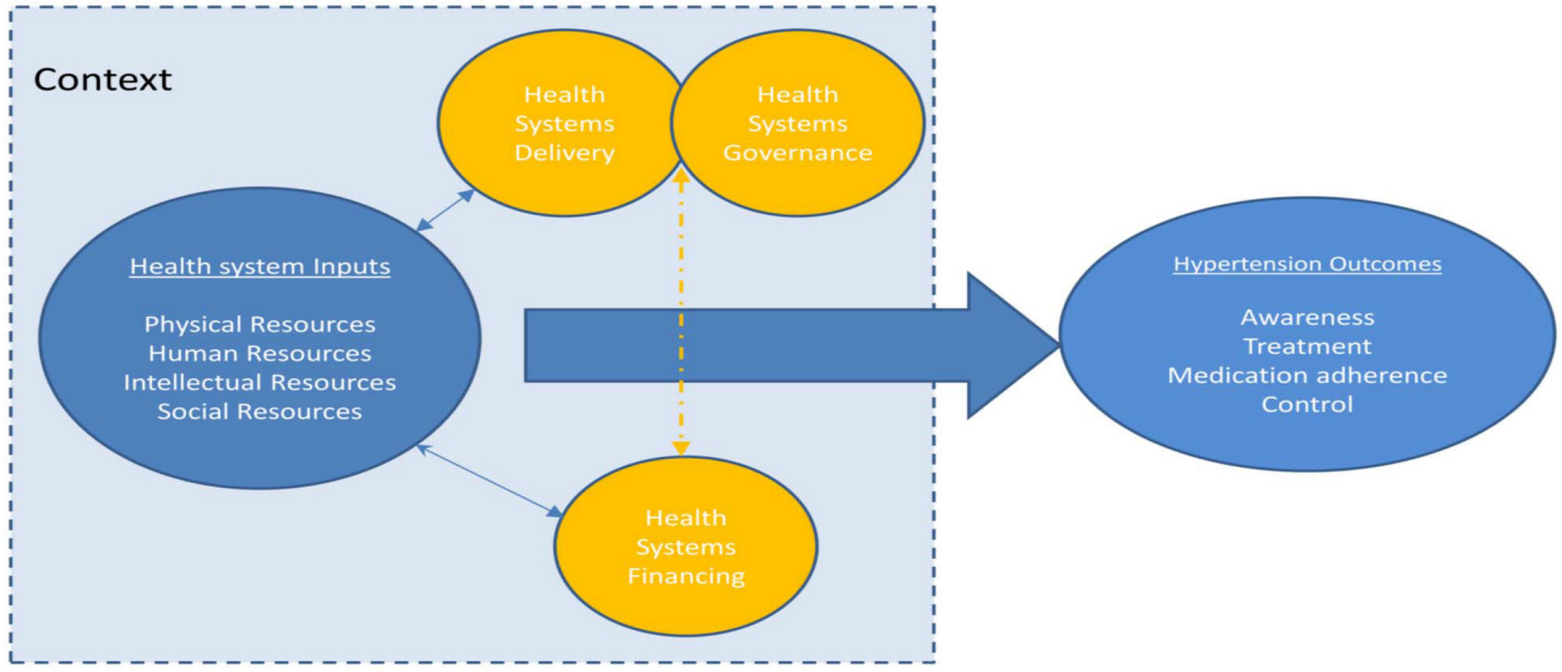
Health Systems Schematic Diagram. Adapted from [4].

Searches of articles were conducted on the PUBMED database. The search was conducted for the period from January 2013 to September 2014. We used the search terms (see Table 1 and Appendix) “*hypertension*” and “*Sub-Saharan Africa”* or “” or “*blood pressure*” and “*Africa*” in combination with factors we considered as “*elements of the health systems”*“ (See Fig 1 and S1 Research Protocol for more details). The review methodology was also adapted from other reviews focused on the influence of health systems on hypertension outcomes [4] which we defined as hypertension awareness, treatment, medication adherence, and control (the achievement of BP below 140/90mm Hg). We also retrieved articles from the reference lists of available studies.

**Table 1:** Search Strategy.

Sub-Saharan Africa	Angola, Benin, Botswana, Burkina Faso, Burundi, Cameroon, Cape Verde, Central African Republic, Chad, Comoros, Congo (Brazzaville), Congo (Democratic Republic), Côte d'Ivoire, Djibouti, Equatorial Guinea, Eritrea, Ethiopia, Gabon, The Gambia, Ghana, Guinea, Guinea-Bissau, Kenya, Lesotho, Liberia, Madagascar, Malawi, Mali, Mauritania, Mauritius, Mozambique, Namibia, Niger, Nigeria, Rwanda, Sao Tome and Principe, Senegal, Seychelles, Sierra Leone, Somalia, South Africa, Sudan, Swaziland, Tanzania, Togo, Uganda, Western Sahara, Zambia, Zimbabwe
Hypertension or high blood pressure adapted from [4]	Hypertension, hypertension awareness, hypertension treatment, hypertension medication adherence hypertension control, high blood pressure.
Health systems factors adapted from [4]	Health facilities, diagnostic equipment, health care workers, treatment guidelines, health insurance, financing, governance, social capital

### Developing a narrative synthesis

We followed the steps delineated by Popay and colleagues[27], namely (1) developing (and/or) identifying a theoretical model, (2) developing a preliminary analysis, (3), exploration of relationships in the data, and (4), assessment of the robustness of the synthesis. Rather than developing a theoretical model as outlined Popay [27], we built on the schematic model developed by Maimaris et al.[4] which consists of four domains relating to key system level inputs that are required for effective management of chronic diseases such a hypertension (See Fig 1). Specifically, *“the model aims to capture the complex interactions and inter-relationships that exist between the elements within a health system*. *It also highlights the important role that context plays in shaping the relationships between health system inputs and outcomes*, *while recognizing the complex adaptive nature of health systems* [4].*”* Preliminary analysis of the available literature consisted of extracting the descriptive characteristics of retrieved articles in a table so as to produce a textual summary of the results. This enabled the exploration of relationships both within and between the studies reviewed as well as quality appraisal of the methodology used in the studies.

### Preliminary Synthesis (Inclusion and exclusion criteria)

We included studies conducted from 1992 to June 2013 that reported the effects of health systems factors on hypertension control in SSA. The year 1992 was chosen as it was the year the first comprehensive evaluation of hypertension burden was conducted in the region [28]. To be included, and using the conceptual model as a guide, studies had to focus on key health system level inputs (See Fig 1) influencing hypertension management defined as: hypertension awareness, treatment, control, and medication adherence. Studies were included in the review if they met the following criteria; 1) distinctly identifiable study conducted in sub-Saharan Africa; 2) A direct measurement of any hypertension outcomes and aspects of health systems; 3) Studies conducted with a distinct population (patients, health care workers etc), populations on treatment, and /or with specific co-morbidities such as diabetes. All titles and abstracts were initially screened by two authors to determine eligibility. Disagreements were resolved by a third reviewer with expertise in hypertension management and the role of health systems. We excluded review articles, book chapters and dissertations. Also, we did not apply any language restrictions and the abstracts of articles conducted in other languages were initially scanned for relevance and translated to English if matching the inclusion criteria.

### Data Extraction and Exploration of the Relationships between and within Studies

Using a standard template, the first author extracted the following information from available studies: (1) publication citation; (2) country where study was conducted and description of study participants; (3) study design; (4) description of hypertension outcome; (5) and aspects of health care system explored. Extracted data was reviewed by the remaining authors for disparities, erroneous and/or inconsistent data. Relationships between and within studies were explored further through thematic analysis to identify emerging themes relative to health systems building blocks and hypertension outcomes.

### Quality Appraisal; Assessing the robustness of synthesis

The quality of the reviewed papers was assessed by two reviewers (JI and CE) using a checklist adapted from a previously described assessment criteria [29]. On the basis of the criteria, each reviewed paper received a quality grade of low, medium, or high, and any disagreement resulted in joint review of the article. The reviewers specifically appraised the data for the following:

1. Question or objective sufficient described?
2. Study design evident and appropriate?
3. For Quantitative Studies-Method of subject/comparison group selection or source of information/input variables described and appropriate? Subject (and comparison group, if applicable) characteristics sufficiently described? For Qualitative Studies-Context of the Study is Clear?
4. For Quantitative Studies-Outcome and (if applicable) exposure measure(s) well defined and robust to measurement / misclassification bias? Means of assessment reported?
5. For Qualitative Studies-Connection to a theoretical framework / wider body of knowledge?
6. Sampling strategy described, relevant and justified? Sample size appropriate?
7. Analytic methods described/justified and appropriate?
8. For Quantitative Studies-Some estimate of variance is reported for the main results? Data analysis clearly described and systematic?
9. For Qualitative Studies- Data analysis clearly described and systematic?
10. Results reported in sufficient detail?
11. Conclusions supported by the results?

## Results

**Fig 2:**
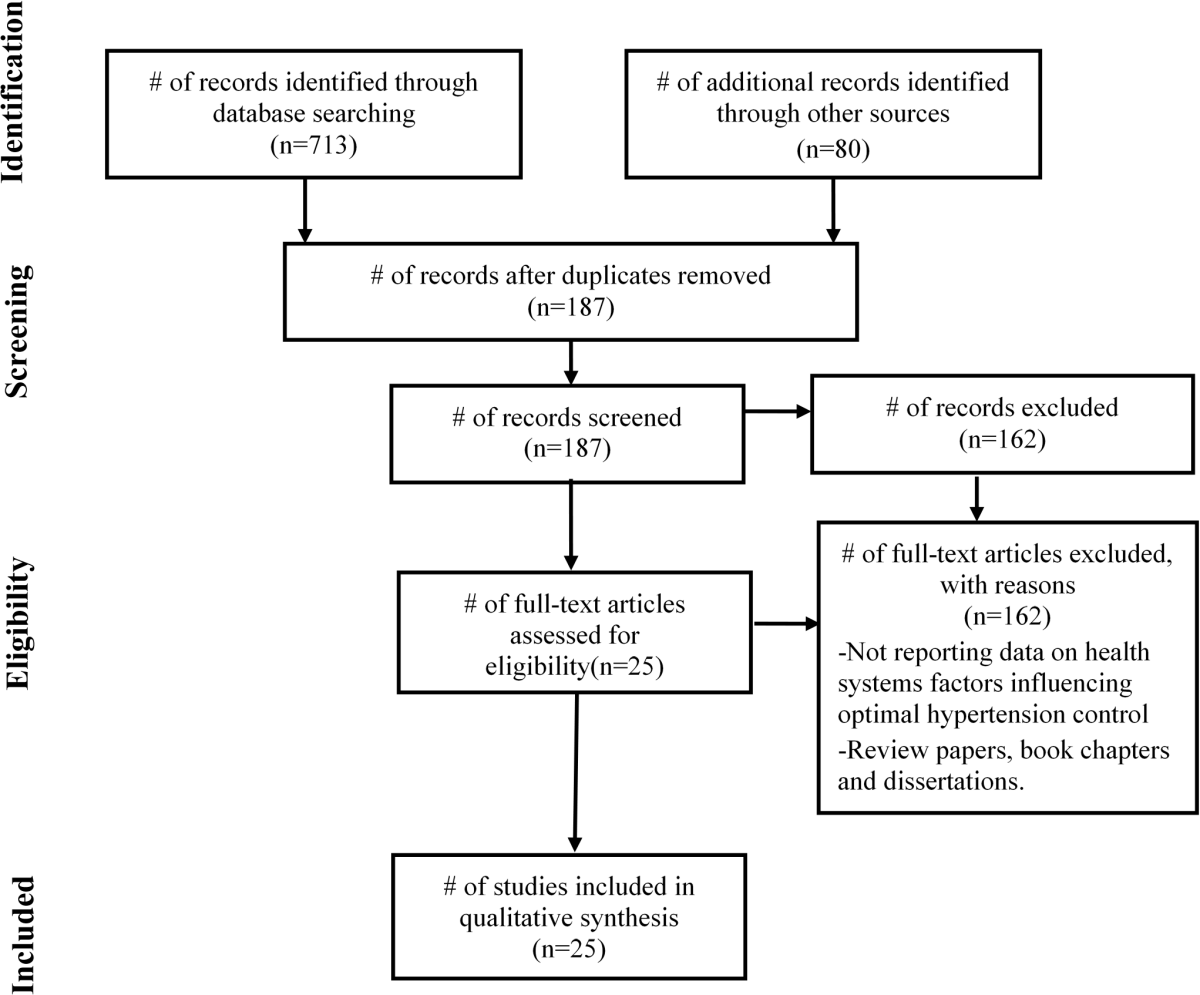
Flow of information through the different phases of the systematic review.

PRISMA guidelines were used to guide reporting of the literature reviewed and a flow diagram is shown in Fig 2 (See S1 PRISMA Checklist for more detail)**.** Seven hundred and ninety-three articles were screened by title and abstract for inclusion. Out of 793 articles, the full text of 187 articles were obtained and assessed for eligibility. 25 studies met the eligibility criteria for this review. Full details of the included studies, including study design, population, setting, key findings and risk of bias assessment can be found in Table 2. 18 studies were quantitative in nature, four qualitative [30–33], and two were mixed-method study [34,35]. Of the 18 studies, one was a cluster-randomized trial [36]; 4 were cohort studies [37–40]; one of which was retrospective [39] alongside 2 other retrospective studies [41,42] and one mixed-method retrospective study [35]; 10 were cross-sectional studies. Majority of the studies were carried out in South Africa (n = 9) and in Nigeria (n = 8), 4 in Cameroon, while the rest in the Democratic Republic of Congo, Ethiopia, Ghana, and Tanzania. The narrative synthesis identified the following themes (Table 3): 1) how physical resources within and outside the health system influence supportive mechanism for optimal hypertension control; 2) the role of human resources with enabling and/or inhibiting hypertension control goals; 3) the availability and/or use of intellectual resources; and 4) how health systems financing facilitate and/or compromise access to products necessary for optimal hypertension control.

**Table 2:** Summary of Literature Reviewed.

Author	Setting and Sample Size	Study Design	Detailed Hypertension-related Findings
Hendriks et al. (2014)	Nigeria, community dwelling adults, n = 3,023	Cohort (24 month follow up)	Hypertension Control: Systolic blood pressure decreased by 10.41 (95% CI, -13.28 to -7.54) mm Hg among patients in the community-based health insurance program (CBHI). Diastolic blood pressure decreased by 4.27 (95% CI, -5.74 to -2.80) mm Hg in the program area
Peck et al., 2014	Tanzania, n = 335 health care workers	Cross-sectional	Hypertension Treatment: First-line drugs for hypertension were frequently lacking. Basic diagnostic equipment for hypertension was consistently present in 63–75% of health facilities, but even hospital outpatient departments sometimes did not have essential instruments such as sphygmomanometers.
Ndou et al., 2013	South Africa, n = 214 patients	Mixed-methods	Hypertension Control by Community Health Workers in 21.4% of Kgatelopele (community-participants) patients (12/56) were controlled at >40% of health checks in comparison to 13.1% of clinic patients (22/168).
Ambaw et al., 2012	Ethiopia, n = 384 participants in Addis Abba	Cross-sectional	Hypertension Treatment: As the distance from the hospital decreased, the adherence to treatment of HTN improved (AOR = 2.02, 95% CI = 1.19, 3.43).
Ilesanmi et al., 2012	Nigeria, n = 250 rural patients	Cross-sectional	Hypertension Treatment: Mean cost of treatment was ₦1440 ± 560 ($9.6 ± 3.7) with 52.8% spending ≥ 10% of their income on treatment.
Mbouemboue et al. (2012)	Cameroon, n = 117 hypertensive patients in Adamawa region	Cross-sectional	Hypertension awareness (baseline 1 for low cost of medications); medium cost 0.35 [0.06–2.07]; high cost 0.44 [0.07–2.75]
Labhardt et al., 2011	Cameroon, n = 221 patients	RCT	Hypertension Control: Among the 104 hypertensive patients retained at 1 year, 72 (69%) had a BP of 140⁄90 mmHg at the last visit. Overall average systolic BP decreased from 175.8 to 135.6 mmHg (95% CI: 35.0–45.4, P< 0.001) and diastolic from 100.7 to 80.1 (17.3–23.9; P<0.001). Financial incentives added relevant costs to the program. The average monthly cost to patients for antihypertensive medication was €1.10 ± 0.9. The average transport cost among these patients was €1 ± 1.
Osamor et al., 2011	Nigeria, n = 440	Cross-sectional	Medication Compliance: 51% of the subjects reported high compliance with antihypertensive medication. Among respondents with low compliance, they perceived antihypertensive medications to be necessary but indicated that costs of medication among other factors hindered compliance. The medications were expensive compared to income, and some participants could only buy the quantity of medication they could afford instead of the full prescription (for example, two weeks instead of four weeks).
Parker et al., 2011	South Africa, n = 16 physicians	Cross-sectional	Hypertension Treatment: Ten (62.5%) of the doctors surveyed aimed to treat hypertension to target, and recommendations on lifestyle modifications were reportedly poorly done. While 11 (68.8%) of the doctors were aware of the South African hypertension guidelines, were (81.8%) of them were not conversant with the contents thereof. Doctors estimated that only 35% of their patients are treated to target.
Labhardt et al., 2010	Cameroon, Central region, n = 493 hypertensive patients with at least one documented follow-up visit at a non-physician clinician facility (NPC)	Cohort study-before and after intervention, no control group	Hypertension Control: Fall in BP from baseline to follow up. the BP-decrease remained significant: -26.5 mmHg (95%CI: -12.5 to -40.5) for systolic and -17.2 mmHg (95%CI: -7.1 to -27.3) for diastolic BP.
Kengne et al. 2009	Cameroon, Yaounde (urban) and Bafut (rural), n = 454 patients in a nurse-led protocol	Cohort, 24 month follow up	Hypertension Control: Between baseline and final visits, systolic and diastolic blood pressures dropped by 11.7 mm Hg (95% confidence interval, 8.9–14.4) and 7.8 (95% confidence interval, 5.9–9.6), respectively (P < .001).
Gombet et al., 2009	Congo, n = 197 patients in Brazzaville	Cross-sectional Retrospective	Hypertension Control: The overall cost of hypertension emergency care ranged from 74.600 to 18.4600 CFA francs (111.90 to 276.90 euros), i.e., a mean per-patient cost of 159.600 +/-44.107 CFA francs (239.40 +/- 66.20 Euros).Most people living in Brazzaville cannot afford hypertensive emergency
Rayner et al., 2009,	South Africa, n = 451 patients	Cross-sectional	Hypertension Control: BP was reduced by 26.4/17.6 mmHg (*p* < 0.001) in 220 patients with a documented initial BP. Co-morbidities were present in 322 (71.4%) patients and overall, 37.9% had more than one co-morbidity. Lifestyle modification was not uniformly applied, with only 46.1, 59.6 and 56.8% receiving advice about weight loss, exercise and diet, respectively
Bradley et al., 2007	South Africa, n = 43 community health workers in Khayelithsha	Qualitative methods	Hypertension Awareness: Lack of knowledge about hypertension and it’s risk factors among community health workers
Dennison et al., 2007	South Africa, n = 403 peri-urban blacks	Cross-sectional	Hypertension Control: No significant effect of provider type on systolic BP control below threshold (>140 mmHg systolic and >90 mmHg diastolic BP); Diastolic BP 3.29 mmHg greater in public versus private
Kagee et al., 2007	South Africa, n = 23 participants from Western Cape.	Qualitative methods	Medication Adherence: Participants noted that financial barriers related to travelling to clinic, missing a day’s wage due to clinic visit, and costs of clinic fees and medication influenced adherence to medication. Also, long waiting times at clinic and long travelling distance.
Thorogood et al., 2007	South Africa, n = 105 participants, Agincourt sub-district	Mixed-methods	Hypertension Treatment and Control: Clinic nurses discussed problems with the availability of drugs in the clinics. They either had to deny treatment to patients or switch the treatment to another drug, both actions that are likely to reduce adherence to medication. Nurses also said that they were often unable to monitor blood pressure levels in those with hypertension due to lack of equipment in the clinics and many sphygmomanometers were not functioning at all
Ono et al., 2006	Nigeria, n = 64 patients in southwest region	Cohort (3 year follow up)	Hypertension Treatment: There was a tendency to place patients on monotherapy or "no drug treatment" with successive repeat visits to the clinic, even in cases of uncontrolled systolic blood pressure, as well as declining prescription of moderately aggressive combination therapy as patients revisited the clinic. Isolated systolic hypertension (ISH) patients who received "no drug treatment" on occasions after enrollment were either in borderline stage 1 ISH (33.3%) or in stage 1 ISH (66.7%).
Yusuf et al., 2005	Nigeria, n = 200 patients, in Ibadan	Cross-sectional Retrospective	Medication Adherence: Patient adherence with therapy was documented as adequate in 77.5% (107) of cohort. Adverse drug reactions were documented, by physicians, in only 10.9% (15) of cohort. There appear to be currently no organized institutional adverse drug reaction monitoring, detection and documentation system in place.
Buabeng et al., 2004	Ghana, n = 128 patients at the Komfo Anokye Teaching Hospital	Qualitative methods	Medication Adherence: 93% of the interviewed patients did not comply with their medications. 96% of the non-compliant patients cited unaffordable drug prices as the main reason for non-compliance.
Mendis et al., 2004	Nigeria, n = 1000 hypertensive patients attending. 56 randomly selected primary- (n = 42) and secondary-level (n = 2) health-care and private health-care (n = 12) facilities.	Cross-sectional	Hypertension Awareness, Treatment, and Control: Laboratory and other investigations to exclude secondary hypertension or to assess target organ damage were not available in the majority of facilities, particularly in primary care. A considerable knowledge and awareness gap related to hypertension and its complications was found, both among patients and health-care providers. Blood pressure control rates were poor (28% with systolic blood pressure (SBP) < 140 mmHg and diastolic blood pressure (DBP) < 90 mmHg] and drug prescription patterns were not evidence based and cost effective. The majority of patients (73%) in this low socio-economic group (mean monthly income 73 US dollars) had to pay fully, out of their own pocket, for consultations and medications.
Salako et al., 2003	Nigeria, n = 143 patients,	Cross-sectional	Hypertension Treatment and Control: 51 (36%) of the subjects described as being fully controlled on the treatment instituted while 54 (38%) of the subjects were not controlled at all. In about 18% of the patients, the systolic blood pressure alone was controlled while in 8% the diastolic blood pressure alone was controlled. Furthermore, level of blood pressure control in this study is poor suggesting that availability of free drug alone is not enough to improve adherence to antihypertensives.
Daniels et al., 2000	South Africa, N = 4 community health care centers, 15 physicians and 10 nurses IN Western Cape	Qualitative methods	Hypertension Treatment: Treatment guidelines were not systematically implemented at local CHCs and individual doctors consulted the guidelines infrequently. Several themes were identified as barriers to the application of the guidelines, including the consultation process by which the guidelines were developed, time constraints, skepticism about durability of the guidelines, conflict with local practices, health system problems, and patient beliefs.
Steyn et al., 1999	South Africa, n = 202 patients in Cape town	Cross-sectional	Hypertension Control: 41.6% had a BP above 160/95 mm Hg and only 42.1% had a BP below 140/90 mm Hg. Patients had little knowledge of either the consequences of hypertension or the actions needed to ensure that complications were prevented; 31% suggested home remedies for hypertension. The majority of the patients were satisfied with the service they received, but 47% complained about long waiting times, 37% felt that the doctor did not examine them adequately, and 15.5% reported that insufficient medication was provided when filling prescriptions.
Olubodun et al., 1995	Nigeria, n = 42 physicians	Cross-sectional	Hypertension Control: Over half do not usually measure the blood pressure (BP) of all new patients. A third do not investigate before starting therapy. Over half commence drug therapy with less than three BP readings, while over two thirds do so from appropriate BP levels.

**Table 3:** Themes generated from reviewed literature.

Health Systems Framework	Health Systems Factor being investigated	Number of Studies and Citation	Settings of Study
Physical Resources	Unreliable drug supply	3 [34,43]	South Africa, Tanzania
	Longer waiting times	2 [33,57]	South Africa
	Equipment	3 [34,43, 47]	Nigeria, South Africa, Tanzania
	Poor knowledge/referral/documentation	5 [31, 41, 43, 52, 58]	Nigeria, South Africa, Tanzania
	Long traveling distance	2 [33, 44]	Ethiopia, South Africa
Human Resources	Task-shifting to non-physician clinicians	4 [35, 36, 38, 40]	Cameroon, South Africa
	Physician inertia	4 [35, 39. 45. 46]	Nigeria, South Africa
	Failure to intensify medication	1 [39]	Nigeria
Intellectual Resources	Availability and/or use of treatment guidelines	4 [30, 41, 43, 45]	Nigeria, South Africa
Health Systems Financing	Medication co-payments/cost of clinic visit	6 [32, 33, 42, 48, 49, 51]	Cameroon, Congo, Ghana, Nigeria, South Africa
	Health insurance coverage	1 [33]	South Africa
	Free anti-hypertensive medications	2 [37, 50]	Nigeria

### Physical Resources

The findings identified how physical resources both within and outside the health care system influenced effective management of hypertension in region [4]. The physical resources operating within health systems include; unreliable drug supply, longer waiting times for appointment, the lack of functioning equipment, and poor referral and/or documentation of hypertension management. In Tanzania, Peck and colleagues [43] observed that first line drugs for hypertension were lacking and “*even hospital outpatient departments sometimes did not have essential instruments such as sphygmomanometers*.*”* In the Agincourt sub-district of South Africa, Thorogood and colleagues [34] found that unreliable drug supply were among the barriers influencing the provision of effective treatment for hypertension. Clinic nurses interviewed noted that *“they either had to deny treatment to patients or switch the treatment to another drug*, *actions that are likely to reduce adherence to medication*.*”* The nurses also reported that “*they were unable to monitor blood pressure levels in those with hypertension due to lack of proper equipment in the clinics and/or non-functioning sphygmomanometers*.*”* In Nigeria, Mendis and colleagues (2004) observed that laboratory and other investigations to exclude secondary hypertension or to assess target organ damage were not available in majority of facilities.

Physical resources operating outside the health system include; distance to clinic, particularly long travelling distance. in northwest Ethiopia, longer distance to hospital was found to be significantly associated with adherence to hypertension treatment recommendations with patients from long distant areas less likely to be adherent as compared to patients who resided closer to hospitals [44]. Finally, participants in a qualitative study in South Africa stated that travelling to the clinic where they received medication was extremely problematic. Coupled with the long travelling distance, absence of public transport and missing a day’s wage, was a major impediment to attending clinic visits regularly [33].

### Human Resources

The findings on human resources (trained health care workers) and optimal management of hypertension in SSA were mixed. For example, studies have found that task-shifting to non-physician clinicians for integrated management of hypertension is feasible and effective with improving hypertension care in SSA. In a cohort study conducted by Kengne and colleagues [38] in rural and urban Cameroon, the authors implemented a nurse-led hypertension management protocol with 454 patients. Between baseline and follow-up, the authors observed that systolic blood pressure and diastolic blood pressure rates dropped by 11.7 mmHg (8.9–14.4) and 7.8mmHg (5.9–9.6) respectively. Similarly, in a study conducted by Ndou and colleagues [35], patients receiving home visits from community health care workers were more likely to achieve blood pressure control goals than those receiving usual care for from the clinic.

On the other hand, physician inertia and/or failure to intensive antihypertensive medication are common in the region. In southwestern Nigeria, Ono and colleagues [39] observed that physicians increasingly “*placed patients on monotherapy or "no drug treatment"*,. *even in cases of uncontrolled systolic blood pressure*.*”* In the Gauteng Province of South Africa, Ndou and colleagues [35] observed that insufficient monitoring of patient outcomes by clinical staff hampered efforts to improve the management of hypertension [35]. Also in South Africa, among physicians interview by Parker and colleagues [45], observed that therapy was not routinely intensified for the majority of patients [45]. Similarly, in a cross-sectional study of blood pressure control in 451 hypertensive patients in South Africa, Rayner and Schoeman [46] found evidence of physician inertia with 60.6% of high-risk patients not at target and only 46.1%, 59.6% and 56.8% receiving advice about weight loss, exercise and diet, respectively. Moreover, the authors noted that in the patients not at target BP, there was no action plan in 22.9%, despite the fact that physician had self-identified these patients not to be at target and the majority of patients were either on one- or two-drug treatment [46].

### Intellectual Resources

Three studies documented the availability and/or use of intellectual resources such as hypertension treatment guidelines. In South Africa, Daniel and colleagues [30], observed ambivalence towards the South African hypertension treatment guidelines noting *“that physicians do not regularly consult the guidelines citing insufficient resources and time*, *overcrowded clinics*, *decreasing staff*, *and few opportunities for continuing medical education (CME)*.*”* Parker and colleagues [45] also observed that in South Africa, physicians were not conversant with the contents of the guidelines. In cases of adverse drug reactions, Yusuf and Balogun [41] observed that there was no *‘institutional system in place to monitor*, *detect and document adverse drug reactions among patients on anti-hypertensive drug therapy*.*’*

### Social resources

No study reported on the impact of social capital on hypertension awareness, treatment and control in SSA.

### Health System Financing

The findings on health systems financing which ranged from medication co-payments, costs of clinic visit to health insurance coverage were also mixed. A recent study among 3,023 community dwelling adults by Hendriks and colleagues [37] found that among patients in the community-based health insurance coverage program, systolic blood pressure decreased by 10.41mmHg (-13.28 to 7.54) and diastolic blood pressure by 4.27mmHg (-5.74—2.80). Despite these promising findings, health insurance coverage is often not available in many settings in SSA. Mendis and colleagues [47] noted that ‘*the fact that most patients have to pay out of their own pocket for consultation and medications either fully or in part*, *is likely to have a negative impact on long-term management of hypertension*. In a survey of 250 rural patients with primary hypertension in South West Nigeria, Ilesanmi and colleagues [48] observed that about half of the patient were spending a tenth or more of their income on health care related expenses. Similarly, in a survey of 440 community residents in southwest Nigeria, participants stated that *“medications were expensive compared to their income and they only bought the quantity of medication they could afford instead of the full prescription (for example*, *two weeks instead of four weeks)”* [49]. High costs of medications have been shown to be associated with poor compliance to antihypertensive treatments in Ghana [32]. Buabeng and colleagues [32] found that in a sample of 128 patients, 93% did not comply with their medications, citing unaffordable drug prices as the main reason for non-compliance. However, one study found that even in cases where people are given access to free antihypertensive medication, hypertension was not controlled in about 38% of participants *“suggesting that availability of free drug alone is not enough to improve adherence to antihypertensives [50].”* A recent study conducted in Cameroon examined the association of medication costs with hypertension awareness and did not find one, although the confidence intervals were wide (odds ratio for hypertension awareness for high medication cost versus low medication cost 0.44, 95% CI 0.07–2.75) [51].

### Governance and Service Delivery

No study examined health system arrangement and hypertension management relative to governance. But studies on physician inertia, such as the study conducted by Dennison and colleagues [52] in South Africa highlighted the influence of service delivery on hypertension management. For example, the authors suggested the following- *“healthcare provider inertia sends a powerful message to patients*, *families*, *and other health care providers that hypertension control is not important*.*”*[52]

## Discussion

The literature reviewed provides evidence on the health systems factors influencing hypertension awareness, treatment, control, and medication adherence in SSA. The findings showed that physical resources coupled with human and intellectual resources alongside health systems financing act in varying ways to influence optimal hypertension management in the region. Although barriers to optimal hypertension control exists at the level of the patients for example, in relation to knowledge about hypertension and its consequences, over and beyond these patient-level barriers [9,53], the findings of the literature reviewed suggest that systems level factors in the form of physical, human, intellectual resources and health systems financing enable and/or hinder equitable access to optimal hypertension care. Systems-level barriers such as lack of finances, high costs of drugs, longer waiting times at health systems, limited capacity for adequate diagnosing and prescribing, poor tracing of non-attending patients, and even poor provision of care by healthcare providers themselves also disproportionately limit the capacity of certain countries in SSA to manage and control increasing hypertension rates in the region.

The ability to provide optimal services by health care workers are influenced by increased patient load, overall increase in workload, and shortage of workers [54]. One key issue is the brain drain that has plagued SSA for over 20 years [55,56]. It has led to an acute and chronic shortage of health workers available to implement primary and secondary prevention of hypertension at available health care systems [54]. Although the literature reviewed provided information on the current state of knowledge regarding human resource that influence optimal management of hypertension, many gaps in the literature remain. For example, few studies examined the current workload of available health care workers or how their compensation rates may influence their ability to provide quality care for optimal hypertension. Moreover, the lack of available health care workers in most health care settings in SSA calls for new strategies to reduce brain drain. This knowledge base is necessary if reduction in hypertension related morbidity and mortality rate in SSA is to be achieved.

The same is obviously true for financial barriers in the form of high costs of medication for hypertension and overall health systems costs. For optimal control of hypertension to be achieved, considerable investments need to be made to improve access to equitable healthcare services without imposing excessive burden to individuals and households in the region. The availability of anti-hypertensive drugs is crucial for long-term treatment and control of hypertension. But studies also indicate that free antihypertensive medication is not sufficient by itself alone without the provision of other services such as the availability and use of accurate blood pressure monitoring devices or blood pressure treatment guidelines. Overall quality of care including acceptability (shorter waiting times), affordability (health insurance coverage or low clinic costs), accessibility (shorter distance to health care systems) also matters given the potential for low utilization of health care systems for adequate management of hypertension or non-compliance with antihypertensive drug therapy.

Taken together, delineating the health systems factors influencing optimal hypertension control in the region is a necessary first step towards the development and implementation of interventions aimed at mitigating the systems-level barriers to optimal hypertension control. It is also necessary for researchers to accurately capture the complexities inherent in resource-limited health care systems in SSA and how this may have anticipated or unanticipated effects with hypertension awareness, treatment, control, and medication adherence in the region. While the reduction of hypertension in SSA is influenced by multiple factors operating at the health systems level, identifying key leverage points whether at the level of physical, human, intellectual or health systems financing level (instead of targeting only one level), is likely to produce larger and longer lasting effects.

### Limitations of research

There are several limitations for the reviewed research. First, it is impossible to assess a direct link between health systems variables and the increasing prevalence of hypertension in the region because most of the available data are cross-sectional in nature. Longitudinal studies are recommended to understand the full impact of health systems on the management and control of hypertension in the region. Another important issue considered is that objectives of the available literature including the sampling methodology used were highly variable which in turn may raise concerns about the quality of the studies or the logic of combining the findings of all the studies reviewed. The use of narrative synthesis instead of a quantitative, meta-analytic review is crucial as it provided the opportunity to use words and text to summarize and explain findings from the literature reviewed thus providing evidence on the health systems factors influencing optimal hypertension in region. Furthermore, given that the findings were synthesized across countries with few countries represented in this review, the potential for regional differences within SSA may exist with health systems factors associated with optimal hypertension control varying significantly both within and between countries. However, this is a first attempt to discuss the context-specific complexities inherent in certain resource-limited health systems in SSA and how these might influence attempts to mitigate the increasing prevalence of hypertension in region. While a key challenge involves developing ways to understand and account for this complexity in a way that makes intervention practice effective, future research with key stakeholders that utilizes systems thinking strategies such as concept mapping for public health research or systems dynamic modelling, will enrich understanding and elucidate the multiple pathways through which health systems influence optimal management and control of hypertension in the region.

## Conclusion

This narrative synthesis represents an effort to summarize existing data on the health systems factors that influence optimal hypertension control in SSA. The findings illustrate how physical, human, intellectual resources alongside health systems financing interact to influence optimal management and control of hypertension. While there were several methodological shortcomings identified, given the increasing prevalence of hypertension in SSA, the findings of this review underscore the need for conducting much-needed research as well as developing and implementing new interventions on a topic of vital public health significance to the region. Understanding health systems factors is also relevant for understanding the patient-level and/or provider-level barriers that influence awareness, treatment, control, and medication adherence is the region. Furthermore, while there have been significant research effort to explore patient-level or provider-level factors, the success of new interventions for optimal hypertension control will also rely on an understanding of factors operating at the health systems level. Simply put, research or new interventions to reduce the increasing prevalence of hypertension in the region might be futile if the same vigor is not applied to addressing the health systems factors that influence the burden of hypertension in SSA. Moreover, knowledge of these factors have implications for the design and implementation of cost-effective interventions aimed at achieving optimal hypertension control in the region while facilitating the reduction of hypertension-related morbidity and mortality rates in SSA. This paper serves as a necessary first step towards open discussions on the complexities inherent in resource-limited heath systems in sub-Saharan Africa and how that might influence management and control of the increasing prevalence of hypertension in the region.

## Supporting Information

S1 PRISMA Checklist PRISMA checklist. (DOC)

S1 Research Protocol Research Protocol. (DOC)

## References

[1] Ogah OS, Rayner BL (2013) Recent advances in hypertension in sub-Saharan Africa. Heart. 10.1136/heartjnl-2012-303227 23708775

[2] Rayner B (2010) Hypertension: detection and management in South Africa. Nephron Clinical Practice 116: c269–c273. 10.1159/000318788 20639673

[3] Betancourt JR, Carrillo JE, Green AR (1999) Hypertension in multicultural and minority populations: Linkin communication to compliance. Current Hypertension Reports 1: 482–488. 10981110 10.1007/BF03215777

[4] Maimaris W, Paty J, Perel P, Legido-Quigley H, Balabanova D, Nieuwlaat R, et al (2013) The influence of health systems on hypertension awareness, treatment, and control: a systematic literature review. PLoS medicine 10: e1001490 10.1371/journal.pmed.1001490 23935461 PMC3728036

[5] Dzudie A, Kengne A, Muna W, Ba H, Menanga A, Kouam CK, et al (2011) Prevalence, awareness, treatment and control of hypertension in a self-selected sub-Saharan African urban population: a cross-sectional study. BMJ open 2: 17–17. 10.1136/bmjopen-2012-001217 PMC3433777 22923629

[6] Ngoungou EB, Aboyans V, Kouna P, Makandja R, Ecke Nzengue JE, Allogho CN, et al (2012) Prevalence of cardiovascular disease in Gabon: a population study. Archives of cardiovascular diseases 105: 77–83. 10.1016/j.acvd.2011.12.005 22424325

[7] Hendriks M, Wit F, Roos M, Brewster L, Akande T, de Beer IH, et al (2011) Hypertension in sub-Saharan Africa: cross-sectional surveys in four rural and urban communities. PloS one 7: e32638–e32638. 10.1371/journal.pone.0032638 PMC3299675 22427857

[8] Musinguzi G, Nuwaha F (2013) Prevalence, awareness and control of hypertension in Uganda. PloS one 8: e62236 10.1371/journal.pone.0062236 23614041 PMC3629133

[9] Odedosu T, Schoenthaler A, Vieira DL, Agyemang C, Ogedegbe G (2012) Overcoming barriers to hypertension control in African Americans. Cleve Clin J Med 79: 46–56. 10.3949/ccjm.79a.11068 22219234

[10] Iwelunmor J, Airhihenbuwa CO, Cooper R, Tayo B, Plange-Rhule J, Adanu R, et al (2014) Prevalence, determinants and systems-thinking approaches to optimal hypertension control in West Africa. Globalization and Health 10: 42 10.1186/1744-8603-10-42 24886649 PMC4046625

[11] Ali MK, Rabadán-Diehl C, Flanigan J, Blanchard C, Narayan KV, Engelgau M, et al (2013) Systems and capacity to address noncommunicable diseases in low-and middle-income countries. Science translational medicine 5: 181cm184-181cm184. 10.1126/scitranslmed.3005121 23596201

[12] Kalk A, Groos N, Karasi JC, Girrbach E (2010) Health systems strengthening through insurance subsidies: the GFATM experience in Rwanda. Tropical Medicine & International Health 15: 94–97. 19917038 10.1111/j.1365-3156.2009.02424.x

[13] Rispel L, Barron P (2010) Can disease control priorities improve health systems performance in South Africa? SAMJ: South African Medical Journal 100: 801–806. 21414267 10.7196/samj.4439

[14] Mills A, Ataguba JE, Akazili J, Borghi J, Garshong B, Makawia S, et al (2012) Equity in financing and use of health care in Ghana, South Africa, and Tanzania: implications for paths to universal coverage. The Lancet 380: 126–133. 10.1016/S0140-6736(12)60357-2 22591542

[15] Webster J, Kayentao K, Diarra S, Diawara SI, Haiballa AA, Doumbo OK, et al (2013) A Qualitative Health Systems Effectiveness Analysis of the Prevention of Malaria in Pregnancy with Intermittent Preventive Treatment and Insecticide Treated Nets in Mali. PloS one 8: e65437 10.1371/journal.pone.0065437 23843941 PMC3701011

[16] Mbacke CS (2013) African leadership for sustainable health policy and systems research. BMC Health Services Research 13: S15 10.1186/1472-6963-13-S2-S15 23819457 PMC3668247

[17] Mutale W, Chintu N, Amoroso C, Awoonor-Williams K, Phillips J, Baynes C, et al (2013) Improving health information systems for decision making across five sub-Saharan African countries: implementation strategies from the African Health Initiative. BMC Health Services Research 13: S9 10.1186/1472-6963-13-S2-S9 23819699 PMC3668230

[18] Adair-Rohani H, Zukor K, Bonjour S, Wilburn S, Kuesel AC, Hebert R, et al (2013) Limited electricity access in health facilities of sub-Saharan Africa: a systematic review of data on electricity access, sources, and reliability. Global Health: Science and Practice 1: 249–261. 10.9745/GHSP-D-13-00037 PMC4168575 25276537

[19] IJsselmuiden C, Marais D, Becerra-Posada F, Ghannem H (2012) Africa's neglected area of human resources for health research-the way forward. SAMJ: South African Medical Journal 102: 228–233. 22464504

[20] McCord GC, Liu A, Singh P (2013) Deployment of community health workers across rural sub-Saharan Africa: financial considerations and operational assumptions. Bulletin of the World Health Organization 91: 244–253b. 10.2471/BLT.12.109660 23599547 PMC3629450

[21] De Savigny D, Adam T (2009) Systems thinking for health systems strengthening: World Health Organization.

[22] Mounier-Jack S, Griffiths UK, Closser S, Burchett H, Marchal B (2014) Measuring the health systems impact of disease control programmes: a critical reflection on the WHO building blocks framework. BMC public health 14: 278 10.1186/1471-2458-14-278 24666579 PMC3974593

[23] Frenk J (2010) The global health system: strengthening national health systems as the next step for global progress. PLoS medicine 7: e1000089–e1000089. 10.1371/journal.pmed.1000089 20069038 PMC2797599

[24] Reich MR, Takemi K (2009) G8 and strengthening of health systems: follow-up to the Toyako summit. The Lancet 373: 508–515. 10.1016/S0140-6736(08)61899-1 19150128

[25] Meessen B, Hercot D, Noirhomme M, Ridde V, Tibouti A, Tashobya C, et al (2011) Removing user fees in the health sector: a review of policy processes in six sub-Saharan African countries. Health policy and planning 26: ii16–ii29. 10.1093/heapol/czr062 22027916

[26] Schneider H, Blaauw D, Gilson L, Chabikuli N, Goudge J (2006) Health systems and access to antiretroviral drugs for HIV in Southern Africa: service delivery and human resources challenges. Reproductive health matters 14: 12–23. 16713875 10.1016/S0968-8080(06)27232-X

[27] Popay J, Roberts H, Sowden A, Petticrew M, Arai L, Rodgers M, et al. (2006) Guidance on the conduct of narrative synthesis in systematic reviews. Swindon; ESRC methods programme Version 1. Retrieved from http://www.lancaster.ac.uk/shm/research/nssr/research/dissemination/publications/NS_Synthesis_Guidance_v1.pdf

[28] Kaufman J, Barkey N (1992) Hypertension in Africa: an overview of prevalence rates and causal risk factors. Ethnicity & disease 3: S83–101. 8087029

[29] Kmet LM, Lee RC, Cook LS (2004) Standard quality assessment criteria for evaluating primary research papers from a variety of fields: Alberta Heritage Foundation for Medical Research. Retreived from http://www.ihe.ca/documents/HTA-FR13.pdf

[30] Daniels A, Biesma R, Otten J, Levitt NS, Steyn K, Martell R, et al (2000) Ambivalence of primary health care professionals towards the South African guidelines for hypertension and diabetes. S Afr Med J 90: 1206–1211. 11234651

[31] Bradley HA, Puoane T (2007) Prevention of hypertension and diabetes in an urban setting in South Africa: participatory action research with community health workers. Ethn Dis 17: 49–54. 17274209

[32] Buabeng KO, Matowe L, Plange-Rhule J (2004) Unaffordable drug prices: the major cause of non-compliance with hypertension medication in Ghana. J Pharm Pharmaceut Sci 7: 350–352. 15576016

[33] Kagee A, Le Roux M, Dick J (2007) Treatment Adherence among Primary Care Patients in a Historically Disadvantaged Community in South Africa A Qualitative Study. Journal of Health Psychology 12: 444–460. 17439995 10.1177/1359105307076232

[34] Thorogood M, Connor MD, Hundt GL, Tollman SM (2007) Understanding and managing hypertension in an African sub-district: A multidisciplinary approach1. Scandinavian Journal of Public Health 35: 52–59. 10.1080/14034950701355411 PMC2830110 17676503

[35] Ndou T, van Zyl G, Hlahane S, Goudge J (2013) A rapid assessment of a community health worker pilot programme to improve the management of hypertension and diabetes in Emfuleni sub-district of Gauteng Province, South Africa. Global health action 6. 10.3402/gha.v6i0.19228 PMC3556684 23364086

[36] Labhardt ND, Balo JR, Ndam M, Manga E, Stoll B (2011) Improved retention rates with low-cost interventions in hypertension and diabetes management in a rural African environment of nurse-led care: a cluster-randomised trial. Trop Med Int Health 16: 1276–1284. 10.1111/j.1365-3156.2011.02827.x 21733046

[37] Hendriks ME, Wit FW, Akande TM, Kramer B, Osagbemi GK, et al (2014) Effect of health insurance and facility quality improvement on blood pressure in adults with hypertension in Nigeria: a population-based study. JAMA Intern Med 174: 555–563. 10.1001/jamainternmed.2013.14458 24534947

[38] Kengne AP, Awah PK, Fezeu LL, Sobngwi E, Mbanya JC (2009) Primary health care for hypertension by nurses in rural and urban sub-Saharan Africa. J Clin Hypertens (Greenwich) 11: 564–572. 19817937 10.1111/j.1751-7176.2009.00165.x PMC8673012

[39] Ono AE, Oyekigho EW, Adeleke OA (2006) Isolated systolic hypertension: primary care practice patterns in a Nigerian high-risk subpopulation. Sao Paulo Medical Journal 124: 105–109. 16878195 10.1590/S1516-31802006000200011 PMC11060354

[40] Labhardt ND, Balo JR, Ndam M, Grimm JJ, Manga E (2010) Task shifting to non-physician clinicians for integrated management of hypertension and diabetes in rural Cameroon: a programme assessment at two years. BMC Health Serv Res 10: 339 10.1186/1472-6963-10-339 21144064 PMC3018451

[41] Yusuff KB, Balogun O (2005) Physicians' prescribing of anti-hypertensive combinations in a tertiary care setting in southwestern Nigeria. J Pharm Pharm Sci 8: 235–242. 16124935

[42] Gombet TR, Ellenga-Mbolla BF, Ikama MS, Ekoba J, Kimbally-Kaky G (2009) [Cost of emergency cardiovascular care at the University Hospital Center in Brazzaville, Congo]. Med Trop (Mars) 69: 45–47. 19499732

[43] Peck R, Mghamba J, Vanobberghen F, Kavishe B, Rugarabamu V, Smeeth L et al (2014) Preparedness of Tanzanian health facilities for outpatient primary care of hypertension and diabetes: a cross-sectional survey. The lancet global health 2: e285–e292. 24818084 10.1016/S2214-109X(14)70033-6 PMC4013553

[44] Ambaw AD, Alemie GA, MW S, Mengesha ZB (2012) Adherence to antihypertensive treatment and associated factors among patients on follow up at University of Gondar Hospital, Northwest Ethiopia. BMC Public Health 12: 282 10.1186/1471-2458-12-282 22490130 PMC3395566

[45] Parker A, Nagar B, Thomas G, Badri M, Ntusi N (2011) Health practitioners' state of knowledge and challenges to effective management of hypertension at primary level. Cardiovascular journal of Africa 22: 186 10.5830/CVJA-2010-066 21881683 PMC3721906

[46] Rayner B, Schoeman HS (2009) A Cross-Sectional Study of Blood Pressure Control in Hypertensive Patients in General Practice (the I-TARGET Study): Cardiovascular Topics. Cardiovascular journal of Africa: 224–227. 19701531 PMC3721771

[47] Mendis S, Abegunde D, Oladapo O, Celletti F, Nordet P (2004) Barriers to management of cardiovascular risk in a low-resource setting using hypertension as an entry point. Journal of hypertension 22: 59–64. 15106795 10.1097/00004872-200401000-00013

[48] Ilesanmi OS, Ige OK, Adebiyi AO (2013) The managed hypertensive: the costs of blood pressure control in a Nigerian town. Pan African Medical Journal 12. PMC3489397 23133696

[49] Osamor PE, Owumi BE (2011) Factors associated with treatment compliance in hypertension in southwest Nigeria. Journal of Health, Population, and Nutrition 29: 619 22283036 10.3329/jhpn.v29i6.9899 PMC3259725

[50] Salako BL, Ajose FA, Lawani E (2003) Blood pressure control in a population where antihypertensives are given free. East Afr Med J 80: 529–531. 15250626 10.4314/eamj.v80i10.8756

[51] Mbouemboue OP, Yiagnigni E, Koona AK, Cacko J, Ndobo P (2012) Determinants of hypertension awareness and treatment among patients under cardiology follow-up in a Cameroonian regional hospital. International Journal of Collaborative Research on Internal Medicine and Public Health 4: 1663–1672.

[52] Dennison CR, Peer N, Steyn K, Levitt NS, Hill MN (2007) Determinants of hypertension care and control among peri-urban Black South Africans: the HiHi study. Ethnicity & disease 17: 484–491. 17985502

[53] Ogedegbe G (2008) Barriers to optimal hypertension control. The Journal of Clinical Hypertension 10: 644–646. 18772648 10.1111/j.1751-7176.2008.08329.x PMC8109930

[54] Mayosi BM (2013) The 10 ‘Best Buys’ to combat heart disease, diabetes and stroke in Africa. Heart. 10.1136/heartjnl-2013-304130 23680892

[55] Pang T, Lansang MA, Haines A (2002) Brain drain and health professionals: a global problem needs global solutions. BMJ: British Medical Journal 324: 499 11872536 10.1136/bmj.324.7336.499 PMC1122434

[56] Hagopian A, Thompson MJ, Fordyce M, Johnson KE, Hart LG (2004) The migration of physicians from sub-Saharan Africa to the United States of America: measures of the African brain drain. Human resources for health 2: 17 15598344 10.1186/1478-4491-2-17 PMC544595

[57] Steyn K, Levitt N, Fourie J, Rossouw K, Martell R, Stander I. (1999) Treatment status and experiences of hypertension patients at a large health center in Cape Town. Ethn Dis 9: 441–450. 10600067

[58] Olubodun JO (1995) Physicians' approach to the management of hypertension in a developing community. Int J Cardiol 51: 193–197. 8522416 10.1016/0167-5273(95)02409-p

