# Supplementary material for: A Narrative Synthesis of the Health Systems Factors Influencing Optimal Hypertension Control in Sub-Saharan Africa
Source: PLoS One. 2015 Jul 15;10(7):e0130193. doi: 10.1371/journal.pone.0130193 (PMC4503432; doi:10.1371/journal.pone.0130193)
Supplement: S1 Research Protocol — (DOC) [file pone.0130193.s002.doc]

# Protocol_S1

**Objective**

To examine the health systems factors influencing optimal management and control of hypertension in sub-Saharan Africa

**Authors**

This study will be conducted primarily by Julet Iwelunmor (PhD) with support from Collins Airhihenbuwa (MPH, PhD), Chizoba Ezepue (MD), and Olugbenga Ogedegbe (MD)

**Reporting**

We will adhere to the PRISMA (Preferred Reporting Items for Systematic Reviews and Meta-Analyses) statement for reporting results.

**Search**

We will systematically search Medline (Pubmed) for relevant articles. We will also index relevant studies from the bibliography of Maimarias et al.

*Sub-Saharan Africa search related terms*:

Angola, Benin, Botswana, Burkina Faso, Burundi, Cameroon, Cape Verde

Central African Republic, Chad Comoros, Congo (Brazzaville) Congo (Democratic Republic), Côte d'Ivoire Djibouti, Equatorial Guinea, Eritrea, Ethiopia Gabon, The Gambia, Ghana, Guinea, Guinea-Bissau, Kenya, Lesotho, Liberia, Madagascar Malawi, Mali, Mauritania, Mauritius

Mozambique, Namibia, Niger, Nigeria, Rwanda, Sao Tome and Principe, Senegal, Seychelles, Sierra Leone, Somalia, South Africa, Sudan, Swaziland, Tanzania, Togo, Uganda, Western Sahara, Zambia, Zimbabwe

*Hypertension outcomes search related terms:*

Hypertension, hypertension awareness, hypertension treatment, hypertension medication adherence hypertension control, high blood pressure.

*Health systems search related terms:*

Health facilities, Health Insurance Coverage, Medication Costs, Diagnostic equipment, Health care workers, Hypertension Treatment guidelines, Governance, Service Delivery, Social Capital

**Eligibility**

Article eligibility will be determined by three key criteria. Studies must contain:

1. A distinctly identifiable study conducted in sub-Saharan Africa
2. A direct measurement of any hypertension outcomes and aspects of health systems
3. Studies conducted with distinct population (patients, health care workers etc), populations on treatment, and /or with specific co-morbidities such as diabetes.

Two reviewers (JI, CE) will independently screen the list of titles, journals, and abstracts to determine relevance of the article. Final selection will be based on the full text of all potentially applicable articles by the two reviewers independently. In cases of disagreement, a third reviewer (OO and COA) will examine such articles. Results will be discussed until reaching consensus among all four reviewers.

**Data Collection**

Relevant data from all eligible studies will be collected by one reviewer (JI) into a designated spreadsheet. These data will be independently cross-checked by a second assessor (CE). Extracted data will include:

- Publication Citation
- Country where study was conducted
- Study description (e.g., study design, setting, year)
- Sample size
- Hypertension outcome measured (awareness, treatment, control, medication adherence)
- Aspects of health care system explored.

**Assessing Risk of Bias**

Detecting the risk of bias is difficult when dealing with literature that is too heterogeneous. In such circumstances, the widely used Kmet standard quality assessment criteria for evaluating primary research papers from a variety of fields is usually an appropriate tool for gauging the quality of the overall body of evidence. Two authors (JI and CE) will independently assess the risk of bias by answering the following questions adapted from Kmet :

1. Question or objective sufficient described?
2. Study design evident and appropriate?
3. For Quantitative Studies-Method of subject/comparison group selection or source of information/input variables described and appropriate? Subject (and comparison group, if applicable) characteristics sufficiently described? For Qualitative Studies-Context of the Study is Clear?
4. For Quantitative Studies-Outcome and (if applicable) exposure measure(s) well defined and robust to measurement / misclassification bias? Means of assessment reported?
5. For Qualitative Studies-Connection to a theoretical framework / wider body of knowledge?
6. Sampling strategy described, relevant and justified? Sample size appropriate?
7. Analytic methods described/justified and appropriate?
8. For Quantitative Studies-Some estimate of variance is reported for the main results? Data analysis clearly described and systematic?
9. For Qualitative Studies- Data analysis clearly described and systematic?
10. Results reported in sufficient detail?
11. Conclusions supported by the results?

To further help determine the overall risk of bias and the quality of the evidence, each reviewed paper will receive a quality grade of low, medium, or high to inform our decision-making. We will enlist the opinions of the remaining co-authors (COA and OO) should any disagreement arise.

References

1. Maimaris W, Paty J, Perel P, Legido-Quigley H, Balabanova D, et al. (2013) The influence of health systems on hypertension awareness, treatment, and control: a systematic literature review. PLoS medicine 10: e1001490.

2. Kmet LM, Lee RC, Cook LS (2004) Standard quality assessment criteria for evaluating primary research papers from a variety of fields: Alberta Heritage Foundation for Medical Research.
